# What Happens in a Moment

**DOI:** 10.3389/fpsyg.2015.01905

**Published:** 2016-01-07

**Authors:** Mark A. Elliott, Anne Giersch

**Affiliations:** ^1^School of Psychology, National University of Ireland GalwayGalway, Ireland; ^2^INSERM U1114, Department of Psychiatry, Fédération de Médecine Translationnelle de Strasbourg, Strasbourg University HospitalStrasbourg, France

**Keywords:** time, psychological moment, perceptual organization, serial processing, Simon effect

## Abstract

There has been evidence for the very brief, temporal quantization of perceptual experience at regular intervals below 100 ms for several decades. We briefly describe how earlier studies led to the concept of “psychological moment” of between 50 and 60 ms duration. According to historical theories, within the psychological moment all events would be processed as co-temporal. More recently, a link with physiological mechanisms has been proposed, according to which the 50–60 ms psychological moment would be defined by the upper limit required by neural mechanisms to synchronize and thereby represent a snapshot of current perceptual event structure. However, our own experimental developments also identify a more fine-scaled, serialized process structure within the psychological moment. Our data suggests that not all events are processed as co-temporal within the psychological moment and instead, some are processed successively. This evidence questions the analog relationship between synchronized process and simultaneous experience and opens debate on the ontology and function of “moments” in psychological experience.

## What happens in a moment

On an experiential level, a perceptual moment is usually defined as what one experiences in the immediate and “specious present,” i.e., a time interval spanning several hundreds of milliseconds (ms) to a second (see Anderson and Grush, 2009, for definition). For example, when listening to a melody, it would correspond to what is presently in mind, including the note played just before and possibly the note expected to immediately follow. Events are thus clearly distinguished in time within the experienced present. However, a shorter interval has been recorded related to the discretization of psychological events. In this paper we will describe the evidence for this shorter interval. We first describe how earlier work led to the idea that events are processed as co-temporal within elementary time windows of 50–60 ms. We then review our data and the literature that challenges this view by showing that events are automatically distinguished in time at shorter asynchronies. This will allow us to discuss the structure that brings about an elementary quantization of perceptual events.

Although not the first to postulate its existence, Brecher was amongst the first to empirically define a psychological moment below 100 ms. In an ingenious set of experiments, Brecher (1932) established the minimal time required for the perceptual separability of two or more events presented repeatedly and in sequence. Importantly, Brecher's estimate was near identical across modalities in healthy adult participants at 55.3 ms for tactile stimulation and 56.9 ms for visual stimulation, with standard deviations of no greater than 1.4 ms across 14 subjects. Also important was the close corroboration of Brecher's empirical estimate with earlier, although difficult to verify, estimates given by Lalanne (1876) and von Baer. von Baer (1864) is believed to have proposed to the Russian Academy of Sciences in St. Petersburg a fundamental quantum of experienced time at 1/18th of a second, deviations from which could allow for the accurate prediction of the life span of the organism. While influential in the development of ideas such as that of “Umwelt” (phenomenal surrounding) proposed by von Uexküll (1957), no reference is made to this topic in proceedings of the St. Petersburg meeting (e.g., Von Baer, 1909).

A more contemporary conceptualization is related to the idea that during this 53–55 ms interval, neural mechanisms are engaged that render two stimuli as the parts of a single event structure. In this context, the interval described here may refer to a minimum number of oscillations (and so maximum interval) required for two or more neurons to form an assembly that allows for the coding of perceptual structure. This idea refers to a theory expressed in the context of literature on oscillatory neural binding (i.e., the neural code believed to be responsible for the neural coding of relations in perceptual structure, see Singer, 1993, 1999). This theory postulates a minimum number of oscillatory cycles between synchronized neurons is required for a synchronized assembly to be statistically separable from a spurious synchronization—and thus treated by the perceptual system as likely to be coding multi-dimensional perceptual structure. Given oscillations in the broad band 30–70 Hz have been shown to be associated with coding perceptual structure, this would entail between 2 and 3 synchronized events are sufficient to signal perceptual structure within a 53–55 ms interval. It must be acknowledged that this estimate should be treated as speculative, though, since estimates of binding oscillations are taken from a variety of species, which may be subject to different perceptual moments as compared to human beings (see Brecher, 1932, for examples). There are, however a number of estimates of simultaneity thresholds and before treating Brecher's moment in greater detail these, and some other moments require consideration: minimum simultaneity thresholds have been estimated from reports of the simultaneity of spatially separate flashes or lines presented in close spatial proximity. Stimuli such as these may be perceived as simultaneous for inter-flash intervals within the range 1–5 ms; only at larger intervals do they yield the perception of successiveness (in this case of apparent motion, see Sweet, 1953; Westheimer and McKee, 1977; Wehrhahn and Rapf, 1992). Other estimates suggest maximum intervals for the perception of simultaneity and by extension minimum time differences in temporal order discrimination (with attendant motion perception) for intervals of between 17 and 44 ms (Exner, 1875). Empirical evidence has accumulated over the last decades showing that temporal order thresholds across modalities reliably lie in the time range between 20 and 60 ms (Pöppel, 1997; Fink et al., 2006; Babkoff and Fostick, 2013), and with some variation as a function of stimulus properties (Wittmann, 2011).

Unlike paradigms relying upon simultaneity judgments to two events presented simultaneously, or with a small asynchrony, Brecher presented his stimuli as a series of paired events and in this series of events, each event consisted of two simultaneous or slightly asynchronous stimuli. This design, while lending greater ecological validity to his estimate leads to the requirement to process temporal relationships—not only between the two stimuli in each event, but also across events within the series. Simultaneity is estimable only if there is a third (or subsequent) event with which events within the simultaneity are perceptually separable (discussed in detail in Elliott et al., 2007). The point here is that “moments” are not only defined by stimuli bound into a simultaneity, but also by the segregation of the simultaneity from other events in past (and future) moments. This leads to a time paradox, i.e., our difficulty to understand how our perception can be both discontinuous, with separable events, and continuous, with a feeling that time flows without interruption with all events related in time. Considering our experience is rarely of events in staccato, the moments that are measured experimentally may not be a description of phenomenal experience itself but of an underlying discretization such as that implied by Brecher and proposed by Stroud (1955), who postulated the existence of 110 ms quanta underlying phenomenal experience. This was a reinterpretation of Allport's (1964) study of perceived simultaneity in terms of the moment as a continuous, running sample of the input (a Traveling Moment Hypothesis) and thus reconciles the ideas of continuity and discontinuity. This is an important idea in the present context as it allows us to be clear that we are not discussing—directly—our experience of duration (aka time perception) or simultaneity or asynchrony. Instead, and as will become clear, we are discussing what we can learn of the discretization of event structure, implicitly (and very likely at a neural level although there exists very little direct data to support this), and how this relates to the experience of events in an uninterrupted, temporal continuity.

At the ceiling of estimates concerned with immediate perceptual experience are perhaps those of Efron (1970a,b), who describes the minimum duration of an experience as of 137 ms. However and because of its dependence on the organization of event structure, Brecher's moment may be an estimate of an upper limit on elementary perceptual integration. This seems plausible by analogy to spatial organization. In this case, binding between separate features is generally held to result from neuronal assemblies formed by the synchronization of contributive neurons via phase alignment of their spiking in bursts of frequency oscillations (reviewed in Singer, 1999). In addition, Duncan and Humphreys (1989) showed that the very early spatial coding guiding activities such as visual search requires the preattentive segregation of target features from distractors, implying that early grouping is partly derived from relational coding across the entire visual scene.

**Figure 1:**
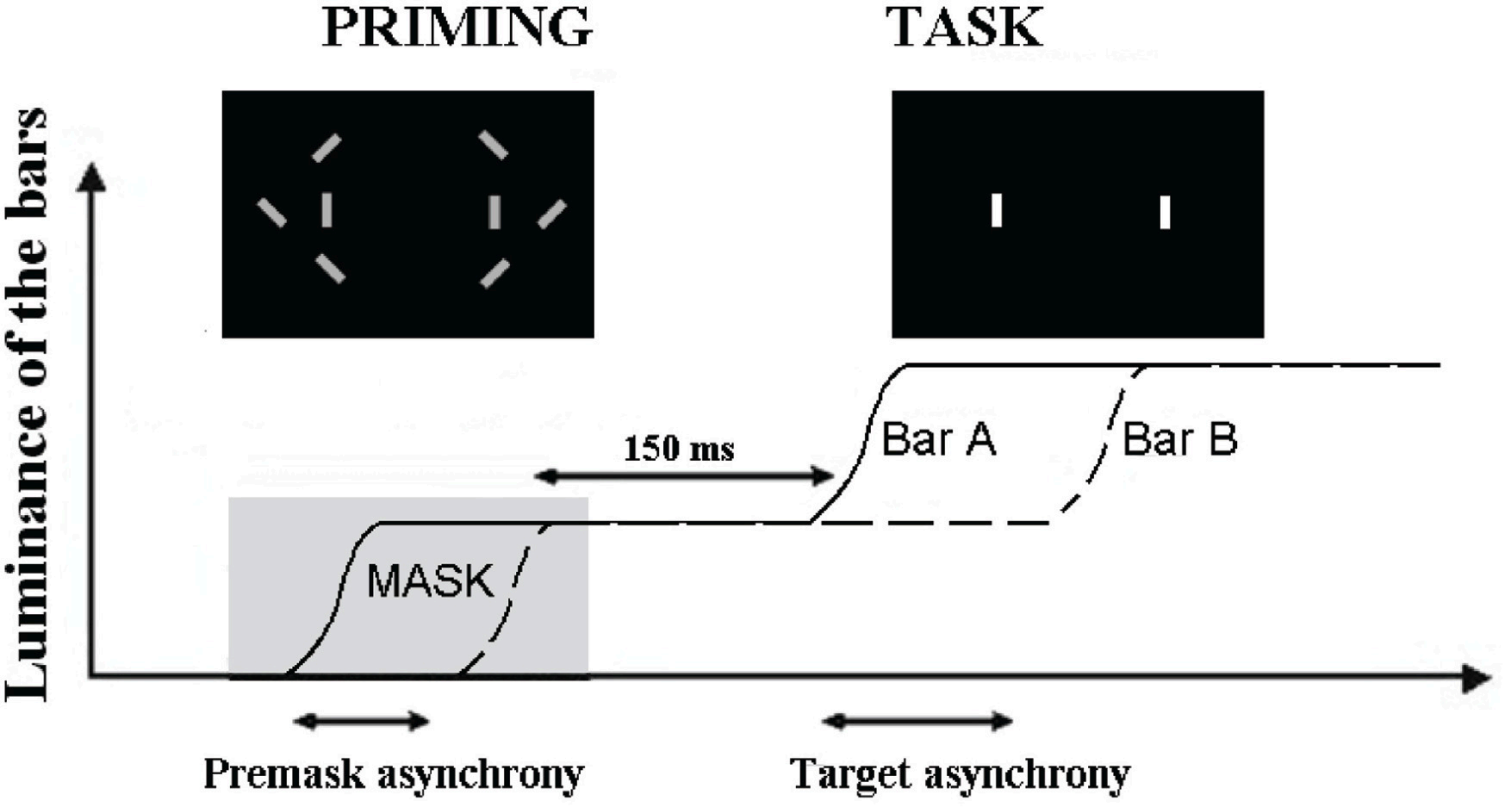
**Illustration of the paradigm used to check for the effect of subthreshold asynchrony**. The curves represent the increase in luminance of the two target bars, A and B. The first increase in luminance is used as a prime and masked by the distracters (“priming” figure). The prime is asynchronous when the two bars increase their luminance asynchronously. The task of the participant is to decide whether the second increase in luminance is simultaneous or asynchronous (reproduced with the permission of Schizophrenia Bulletin).

Using a paradigm similar to that employed by Brecher, in that the paradigm employed repeating visual presentations, Elliott et al. (2007) found mean simultaneity thresholds to target pairings in very close proximity to those reported by Brecher (61 ms). One modification employed by Elliott et al. (2007) was the masked presentation of an asynchrony just prior to target presentation. This allowed investigation of whether a subthreshold synchrony (SB_S_) or asynchronies (SB_A_) would bring about a shift in the threshold for perceived simultaneity, and at which asynchronies, if any, this shift would be found. As illustrated in Figure 1, two stimuli were first presented synchronously (SB_S_) or asynchronously (SB_A_). The asynchrony was made non-detectable by embedding the stimuli within distracters. These stimuli served as primes and, after the disappearance of distracters, they were increased in luminance, following which, participants had to decide whether this luminance increase was simultaneous or asynchronous. Interestingly, SB_S_ and SB_A_ produce different patterns of effects only for targets over a very short range of stimulus-onset asynchronies (SOAs) between targets (including physically simultaneous targets). For target SOAs of up to 21 ms, there appeared to be a small bias towards simultaneity judgments following exposure to SB_S_, relative to simultaneity judgments following exposure to SB_A_. In addition, and for presentations above threshold (61 ms) there seems to be a decreasing tendency to report simultaneity when the targets were preceded by SB_S_. That enhanced simultaneity reportage (following SB_S_) is maintained for SOAs of 0–(14–21) ms is interesting in that the interval 14–21 ms is very close to the maximum separation in time between the firing of different neurons within synchronized neural assemblies in visual cortex (see, e.g., Gray et al., 1989, for data; and Singer, 1993, for review). The rhythmic synchronization of neuronal firing is believed to facilitate the formation of functional neuronal assemblies with those operating in the EEG gamma band (30–70 Hz) associated with functions that includes perceptual processing. What is suggested by the findings of Elliott et al. (2007) is that subthreshold stimulus asynchronies at very short SOAs may influence the efficiency of neuronal synchronization from which we can conclude that functional neuronal assemblies form within the moment with the goal of representing coherent perceptual structure.

The functional moment appears to be constrained by the temporal properties of neurons, i.e., the time needed to synchronize neuron assemblies. In turn, it constrains perception by providing a temporal organization. Since moments are too short to yield a perception of duration (Wittmann, 2011), they are thought to be elementary elements in the composition of trains of thoughts. However, they may not correspond to elementary information: on the contrary and as implied above, they may form as a consequence of information integration. In case of multisensory information, up to 100–200 ms may be needed to distinguish an asynchrony between visual and auditory information (Vatakis and Spence, 2007; van Wassenhove, 2009). Does this mean that perception is a series of snapshots from which we rebuild an experiential continuity a posteriori (Neisser, 1967; Ullman, 1979; Shimojo, 2014; van Rullen et al., 2014)? This possibility requires us to understand both how visual information is correctly organized if it is initially integrated within elementary windows (Gepshtein and Kubovy, 2000), as well as how the coding of discrete moments can be reconciled with our experience of events as in continuous time. Several solutions have been proposed; for instance the overlap of moments (Dainton, 2010). However, there are many different conceptualizations (Phillips, 2014), and recent results may suggest alternative possibilities, which are discussed in the following.

Scharnowski et al. (2009) and Pilz et al. (2013) have found that stimuli perceived as fused in time (i.e., co-temporal) may in fact be initially processed as temporally segregated. In these studies, the authors used stimuli presented in sequence over short time intervals that lead to a temporally fused percept. They applied either TMS (Scharnowski et al., 2009) or masking (Pilz et al., 2013) at different delays to disturb information processing, and examined which of the two successive stimuli dominated the perception. This procedure allowed them to establish that the processing of the two successive stimuli can be disturbed distinctly and in turn. Their results show that disturbance applied 45–90 ms after stimuli onset affects the processing of the first stimulus (leading to the dominance of the second stimulus), whereas disturbance applied 95–420 ms after stimuli onset affects the processing of the second stimulus (leading to the dominance of the first stimulus). It is only after delays of 400–500 ms that both stimuli are perceived as temporally fused with neither TMS nor masking modifying the fusion. These results show that information integration is slow and that perception is more discrete than believed from subjective reports. In addition, the results suggest a specific time course for information processing: for between 400 to 500 ms, successive stimuli are as yet not integrated, and instead processed one after the other, in sequence.

**Figure 2:**
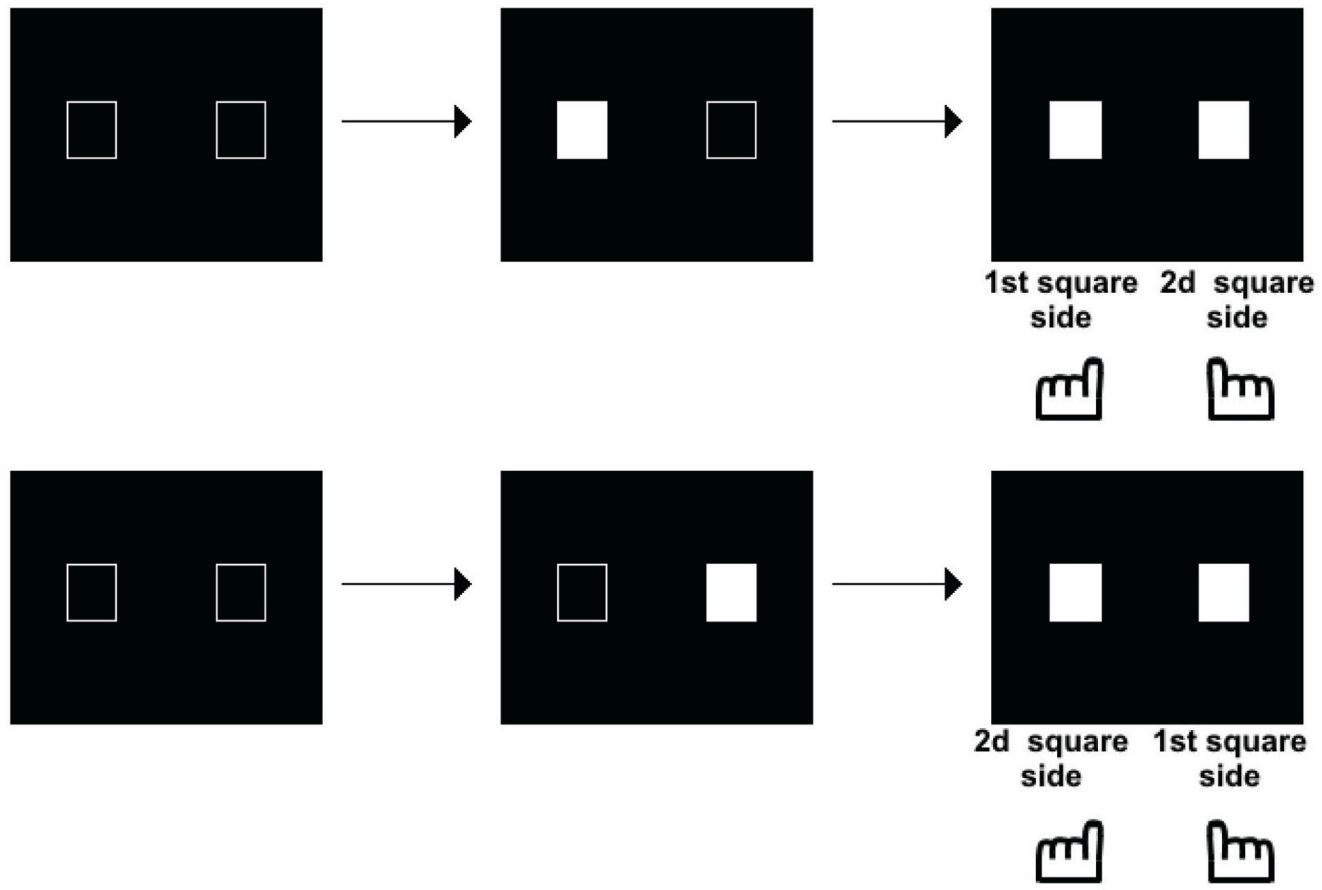
**Illustration of the paradigm used to measure a Simon effect when stimuli are asynchronous**. The asynchrony is manipulated from 0 to 100 ms by 8 or 17 ms steps. The subjects decide whether the stimuli are simultaneous or asynchronous and press a response key accordingly. The Simon effect shows in a tendency to press on either the first or second stimulus side, whatever the side of this stimulus, as shown on the figure.

Such a possibility was explored in another series of studies, initially aimed at exploring the time course of perception in schizophrenia. These studies were motivated by the fact that patients with schizophrenia have been described as suffering from a fragmentation of consciousness, with a loss of the sense of time continuity (Fuchs, 2007). Several studies have shown a lengthening of the perceptual moment: i.e., patients required larger asynchronies than controls to detect asynchronies (Foucher et al., 2007; Giersch et al., 2009; Schmidt et al., 2011; Lalanne et al., 2012a; Martin et al., 2013). This effect was independent of decisional or another non-specific factor (reviewed in Giersch et al., 2013). The lengthening of the perceptual moment became quite large in presence of distracters (Giersch et al., 2009) or in case of multisensory signals (Martin et al., 2013). The integration of information within temporal windows of several 100 ms in patients questioned the way these subjects interact with the environment, especially as it contrasted with their mild pathological state. The implicit processing of stimuli over time was investigated, i.e., the ability to detect asynchronies independent of a conscious judgment. The Simon effect (Simon, 1969) was used to that aim, which corresponds to the tendency to press on the side of a stimulus independent of the task at hand. For example, if the task is to discriminate between squares and circles, and to press respectively on the left and right side in case of a square vs. a circle, subjects will tend to press on the left whenever the stimulus is displayed on the left, even if it is a circle. The mechanisms of this effect are reviewed in Hommel (2011a,b) and van der Lubbe and Abrahamse (2011), and it was used as a tool to examine the automatic processing of stimuli over time. This first required some adaptation of the Simon effect, since two stimuli and not only one, are presented during temporal tasks. As a matter of fact, when two stimuli are simultaneously displayed on the screen, one on the right side and the other on the left side of the screen, responses cannot be biased on either side, since information is perfectly symmetrical. A bias can be observed only in case of an asymmetry between right and left sides, which occurs in case of an asynchrony between the two stimuli. In case of a clear asynchrony, we have shown that subjects are biased to press on the response key located on the side of the second stimulus, whether it is on the left or on the right (Lalanne et al., 2012a,b; illustrated in Figure 2).

**Figure 3:**
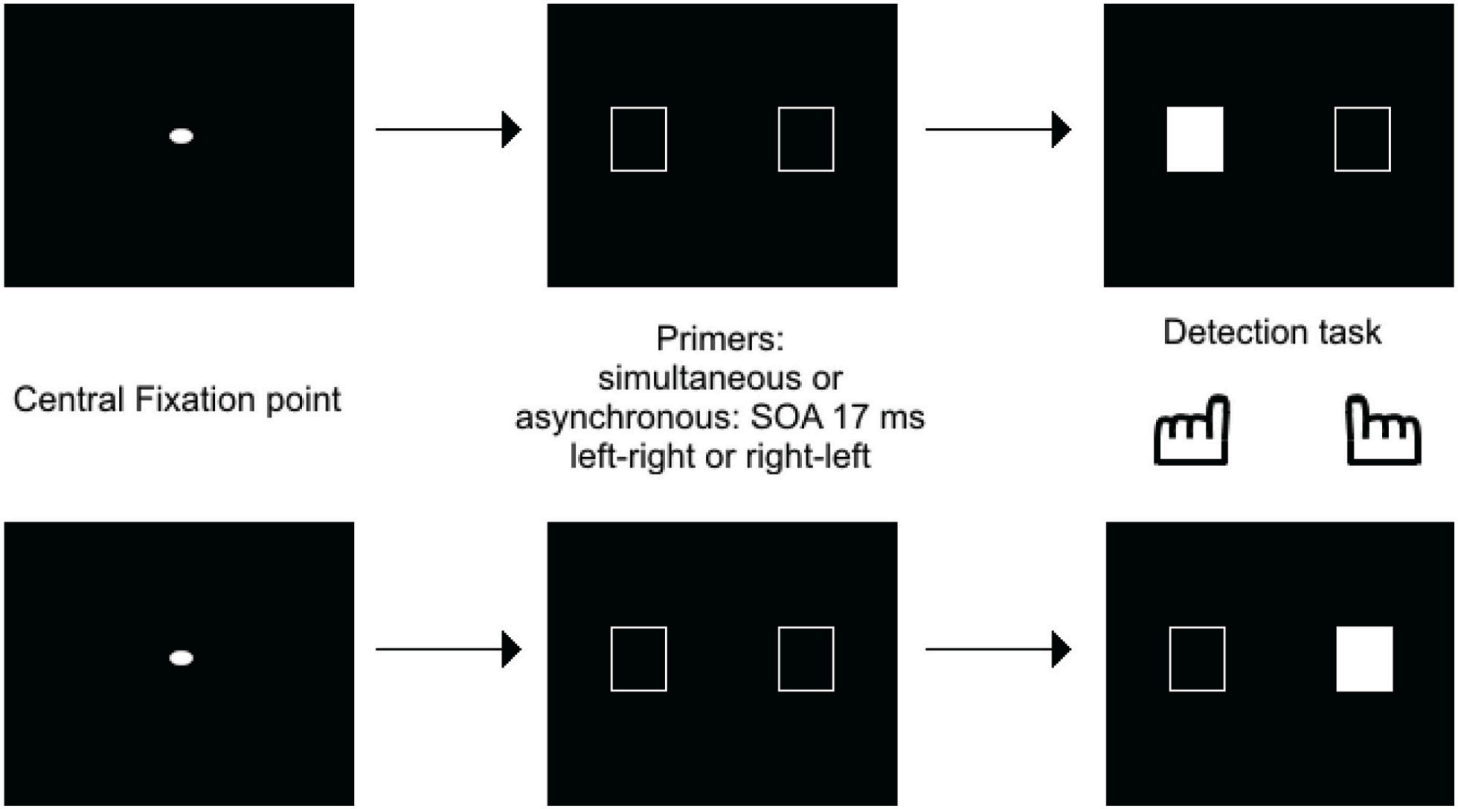
**Illustration of one of the priming paradigms used to test how a 17 ms asynchrony is processed**. Empty frames were used as primes, and were either simultaneous or asynchronous, with a 17 ms asynchrony. After a delay of 100 ms, one of the frame was filled in, representing the target, and subjects had to press on its side. The results showed that RTs were faster when the target was to the side of the 2d rather than the first frame.

This shows that the Simon effect can be used with a simultaneity/asynchrony discrimination task. The critical analysis, however, regarded the exploration of the Simon effect in case of undetected asynchronies, for SOAs below 20 ms. Inasmuch as such asynchronies yield the same amount of “simultaneous” responses as perfect synchrony, they may have been expected to inhibit presentation of a Simon effect. If stimuli are processed as co-temporal, they would indeed yield symmetrical information on both sides on the screen, thus precluding any response bias to either side. This is not what was observed, however. In healthy subjects and for asynchronies as short as 17 ms, a bias to the side of the second stimulus was still observed. Importantly, in patients a bias was also observed to the side of the first stimulus (Lalanne et al., 2012a,b), and this was observed even for asynchronies as short as 8 ms (Giersch et al., 2015). Apart from the significance regarding schizophrenia pathophysiology (Martin et al., 2014), this result is important because it suggests a dissociation between the automatic processing of stimuli over time at delays below 20 ms, and the explicit ability to distinguish events in time. First, patients' ability to detect asynchronies is disturbed, and there is thus a large gap between their ability to explicitly discriminate visual stimuli in time (threshold around 50 ms) and their implicit processing over time (8 ms). Second, the Simon effect is reversed at short (on the side of the first stimulus) and at large asynchronies (on the side of the second stimulus). The implicit ability to discriminate stimuli in time may play a special role in the processing of visual information, by providing the means to follow stimuli over time at an implicit level and with a high temporal accuracy. The possibility that stimuli are processed successively at an unconscious level is suggested by the studies of Scharnowski et al. (2009) and Pilz et al. (2013). Elliott et al. (2007) also suggested that the processing of short asynchronies can be modulated by prior temporal information, i.e., pairs of events whose simultaneity or asynchrony was made non-detectable by the presence of distracters. Asynchronies used to study the time course of information processing (Scharnowski et al., 2009; Pilz et al., 2013) were generally set at around 40 ms, similar to the asynchronies used for primers in Elliott et al. (2007). What the Simon effect at 17 ms brings in addition is evidence that information delayed by asynchronies below 30 ms is not treated as co-temporal by all processes. Specifically, processing, even at small delays is dependent upon stimulus order, suggesting a serialization of processing, even within very short processing windows. This idea is supported by a recent study using healthy volunteers carried out by Poncelet and Giersch (2015). These authors used a priming paradigm to investigate the impact of two (unmasked) primes delayed by 17 ms on the subsequent detection of a target, or on the ordering of two targets (Figure 3).

The aim of this experiment was to check if the primes facilitated or inhibited the detection of a target displayed in the location of the first or second prime^1^. Facilitation would suggest that attentional mechanisms has deployed to the prime location, while inhibition would indicate that attention had shifted from the prime location. This procedure thus allowed examination of how attention shifts as a function of prime presentation. The time course of facilitation and/or inhibition was studied by examining how either evolved over a range of delays between primes and targets. Importantly, asynchronies below 20 ms were non-detectable, and these asynchronies should have led stimuli to be integrated within the same temporal window. Yet, the results confirmed that stimuli presented with asynchronies of 17 ms were processed as temporally separate events. The results also suggested that successive primes were processed serially, consistent with an attentional account. This was indicated by inhibition on the side of the first prime after a short delay (50 ms between primes and target) and by the facilitation on the side of the second prime (100 ms after the occurrence of primes). Importantly, we checked that these effects did not depend on the side of the hand response by changing the response mode (answering on the side of the first vs. second target). Effects are rather a consequence of a shift of attention (see Poncelet and Giersch, 2015, for a more detailed discussion on alternative explanations).

Several studies have now established that visual stimuli are distinguished in time at an implicit level even when belonging to the same perceptual moment. Moreover, it seems healthy observers can unconsciously follow events of the same temporal moment over time, possibly by displacing their attention from one event to the other (Poncelet and Giersch, 2015). All in all these results suggest that information processing is temporally structured even within perceptual moments. It might seem surprising that these effects stayed unnoticed, but this might have been so because most paradigms involve integration processes. For example the flash-lag effect relies on the display of a moving object and a flashing light at some point of the trajectory. Typically, the moving object is perceived ahead of its real location at the time of the flashing light. The shift can correspond to a delay as long as a perceptual moment (25–45 ms; Whitney and Murakami, 1998; Kanai et al., 2004). The implicit processing of information in time should lead to higher precision, but seems not to prevent illusions to occur. This means it is the perceptual moment that shapes conscious experience, even in tasks that do not require an explicit temporal judgment. As a consequence the conscious experience may mask the influence of unconscious mechanisms operating over short delays, i.e., within the perceptual moment. It does not mean, however, that such unconscious mechanisms have no impact on our conscious experience. It only means that this influence is obscured by the operation of other processes, such as postdiction mechanisms (Shimojo, 2014). These help to interpret and render information in the environment as meaningful. In the real world our sensory systems are continuously subjected to multiple, unrelated signals. Under these circumstances, automatically integrating successive events within temporal windows would not help to make sense of this information, while an additional processing step might prove helpful. The availability of individual events before their integration within a perceptual moment, together with a progressive displacement of attention following the events, may be used to apply filters and choose to which extent events will be included within the perceptual moment. This might explain the length of the integration process as described in Scharnowski et al. (2009) and Pilz et al. (2013). Such an hypothesis is speculative and requires confirmation. However, what is clear is that the processing of perceptual information is refreshed at high frequency and that the integration and fusion of information does not preclude access to individual and successive information within perceptual moments.

The high frequency of the information processing refreshment rate converges with the results of Elliott et al. (2007), who has shown that sub-threshold synchrony up to 14–21 ms primes the detection of simultaneous targets. This interval, as suggested above, may correspond to the time required to establish neuronal assemblies of synchronized neurons, and thus to integrate information. This kind of integration, however, would mainly correspond to the binding of single events in time: in this case the presentation of two synchronous or quasi-synchronous stimuli. With reference to the binding of discrete neural processes in ever-changing event structures, we might expand definition of “event structure” to include all events, including the neural events to which the responses of any two functionally separable processes would have to respond. So for example, different neural assemblies are responsible for coding the color and the direction of motion of an object, and their binding ensures the moving item maintains object constancy (i.e., it is perceived as the same object and the same color) in spite of the movement of the object and its spatial displacement, as well as factors such as the observers eye movements (which might include micro-tremor and fast, stimulus-independent oscillations, e.g., Neuenschwander and Singer, 1996). On this basis, relatively fast binding, operating at elementary levels of perceptual processing might be necessary to ensure correct bindings are coded and maintained in spite of unpredictable changes in the event structure to which the synchronized assembly responds. It is only when events are more distant in time, i.e., above 14–21 ms, that assemblies for each event would be distinguished from one another. Such assemblies would be local, and would allow for successive processing of events. However, extracting information on asynchrony and order may require additional processing entailing a comparison of the two events. It is this comparison that would then be accessed consciously, possibly based on longer-range synchronization phenomena. This might be one explanation for the fact that events that are 17 ms apart can be automatically and unconsciously followed in time, based on successive local synchronization phenomena, but that conscious separation of events in time occurs at larger asynchronies only. This possibility is also consistent with the observations that some time is needed to relate successive events with one another, and to integrate them into conscious forms (Scharnowski et al., 2009; Pilz et al., 2013) and across perceptual moments.

The fact that information is automatically distinguished in time within intervals as short as 17 ms is not necessarily in contradiction with the concepts of temporal windows, inasmuch it mainly adds an additional, implicit level of processing. What requires consideration is how evidence for implicit processing changes our understanding of the emergence of the sense of time continuity. The fact that we are able to process and follow information over time with high temporal fidelity seems to contribute to our feeling of time continuity. Indeed, the fact that we can process information with a better time accuracy at an unconscious than at a conscious level means that any environmental change between successive 50 ms windows can be resolved by means of a smoothing over processes responsible for event coding. In addition, each time we consciously look, potentially we unconsciously check for new information several times. In this sense, it can be approximated that we have a continuous access to the outer world.

### Conflict of interest statement

The authors declare that the research was conducted in the absence of any commercial or financial relationships that could be construed as a potential conflict of interest.

## Footnotes

^1^Here the paradigm did not consist in the exploration of the influence of simultaneity/asynchrony on a temporal judgment. The paradigm was built to examine how the successive primers were processed in time.

## References

[B1] Allport D. A. (1964). Phenomenal simultaneity and the perceptual moment hypothesis. Br. J. Psychol. 59, 395–406. 10.1111/j.2044-8295.1968.tb01154.x 5719794

[B2] Anderson H. K. Grush R. (2009). A brief history of time-consciousness: historical precursors to James and Husserl. J. Hist. Philos. 47, 277–307. 10.1353/hph.0.0118

[B3] Babkoff H. Fostick L. (2013). The role of tone duration in dichotic temporal order judgment. Atten. Percept. Psychophys. 75, 654–660. 10.3758/s13414-013-0449-6 23572206

[B4] Brecher G. A. (1932). Die Entstehung und biologische Bedeutung der subjectktiven Zeiteinheit—des Momentes. Z. Vgl. Physiol. 18, 204–243.

[B5] Dainton B. (2010). Temporal consciousness, in The Stanford Encyclopedia of Philosophy, ed Zalta E. N. Available online at: http://plato.stanford.edu/archives/fall2010/entries/consciousness-temporal

[B6] Duncan J. Humphreys G. W. (1989). Visual search and stimulus similarity. Psychol. Rev. 96, 433–458. 10.1037/0033-295X.96.3.433 2756067

[B7] Efron R. (1970a). The relationship between the duration of a stimulus and the duration of a perception. Neuropsychologia 8, 37–55. 10.1016/0028-3932(70)90024-2 5522546

[B8] Efron R. (1970b). The minimum duration of a perception. Neuropsychologia 8, 57–63. 10.1016/0028-3932(70)90025-4 5522547

[B9] Elliott M. A. Shi Z. Surer F. (2007). The effects of subthreshold synchrony on the perception of simultaneity. Psychol. Res. 71, 687–693. 10.1007/s00426-006-0057-3 16614834

[B10] Exner S. (1875). Experimentelle Untersuchungen der einfachsten psychischen Prozesse. Pflugers Arch. 11, 403–432. 10.1007/BF01659311

[B11] Fink M. Ulbrich P. Churan J. Wittmann M. (2006). Stimulus-dependent processing of temporal order. Behav. Process. 71, 344–352. 10.1016/j.beproc.2005.12.007 16413700

[B12] Foucher J. R. Lacambre M. Pham B. T. Giersch A. Elliott M. A. (2007). Low time resolution in schizophrenia lengthened windows of simultaneity for visual, auditory and bimodal stimuli. Schizophr. Res. 97, 118–127. 10.1016/j.schres.2007.08.013 17884350

[B13] Fuchs T. (2007). The temporal structure of intentionality and its disturbance in schizophrenia. Psychopathology 40, 229–235. 10.1159/000101365 17396049

[B14] Gepshtein S. Kubovy M. (2000). The emergence of visual objects in space-time. Proc. Natl. Acad. Sci. U.S.A. 97, 8186–8191. 10.1073/pnas.97.14.8186 10884439 PMC16691

[B15] Giersch A. Lalanne L. Corves C. Seubert J. Shi Z. Foucher J.. (2009). Extended visual simultaneity thresholds in patients with schizophrenia. Schizophr. Bull. 35, 816–825. 10.1093/schbul/sbn016 18359954 PMC2696372

[B16] Giersch A. Lalanne L. van Assche M. Elliott M. E. (2013). On disturbed time continuity in schizophrenia: an elementary impairment in visual perception? Front. Psychol. 4:281. 10.3389/fpsyg.2013.00281 23755027 PMC3664782

[B17] Giersch A. Poncelet P. Capa R. L. Martin B. Duval C. Z. Curzietti M. (2015). Disruption of information processing in schizophrenia: the time perspective. Schizophr. Res. Cogn. 2, 78–83. 10.1016/j.scog.2015.04.002 PMC5609651 29114456

[B18] Gray C. M. König P. Engel A. K. Singer W. (1989). Oscillatory responses in cat visual cortex exhibit inter-columnar synchronization which reflects global stimulus properties. Nature 338, 334–337. 10.1038/338334a0 2922061

[B19] Hommel B. (2011a). The Simon effect as a tool and heuristic. Acta Psychol. 136, 189–202. 10.1016/j.actpsy.2010.04.011 20507830

[B20] Hommel B. (2011b). Attention and spatial stimulus coding in the Simon task: are joinder to van der Lubbe and Abrahamse (2010). Acta Psychol. 136, 265–268. 10.1016/j.actpsy.2010.10.002 21035780

[B21] Kanai R. Sheth B. R. Shimojo S. (2004). Stopping the motion and sleuthing the flash-lag effect: spatial uncertainty is the key to perceptual mislocalization. Vis. Res. 44, 2605–2619. 10.1016/j.visres.2003.10.028 15358076

[B22] Lalanne L. (1876). Sur la duree de la sensation tactile. C. R. Acad. Sci. Paris 90, 1314–1316.

[B23] Lalanne L. Van Assche M. Giersch A. (2012a). When predictive mechanisms go wrong: disordered visual synchrony thresholds in schizophrenia. Schizophr. Bull. 38, 506–513. 10.1093/schbul/sbq107 20876220 PMC3330002

[B24] Lalanne L. van Assche M. Wang W. Giersch A. (2012b). Looking forward: an impaired ability in patients with schizophrenia? Neuropsychologia 50, 2736–2744. 10.1016/j.neuropsychologia.2012.07.023 22842105

[B25] Martin M. Giersch A. Huron C. van Wassenhove V. (2013). Temporal event structure and timing in schizophrenia: preserved binding in a longer “now” Neuropsychologia 51, 358–371. 10.1016/j.neuropsychologia.2012.07.002 22813430

[B26] Martin B. Wittmann M. Franck N. Cermolacce M. Berna F. Giersch A. (2014). Temporal structure of consciousness and minimal self in schizophrenia. Front. Psychol. 5:1175. 10.3389/fpsyg.2014.01175 25400597 PMC4212287

[B27] Neisser U. (1967). Cognitive Psychology. New York: Appleton Century Crofts.

[B28] Neuenschwander S. Singer W. (1996). Long range synchronisation of oscillatory light responses in the cat retina and lateral geniculate nucleus. Nature 379, 728–733. 10.1038/379728a0 8602219

[B29] Phillips I. (2014). The temporal structure of experience, in Subjective Time: The Philosophy, Psychology and Neuroscience of Temporality, eds Arstila V. Lloyd D. (Cambridge MA: MIT Press), 139–158.

[B30] Pilz K. S. Zimmermann C. Scholz J. Herzog M. H. (2013). Long-lasting visual integration of form, motion, and color as revealed by visual masking. J. Vis. 13, 1–11. 10.1167/13.10.12 23962737

[B31] Poncelet P. E. Giersch A. (2015). Tracking visual events in time in the absence of time perception: implicit processing at the ms level. PLoS ONE 10:e0127106. 10.1371/journal.pone.0127106 26030155 PMC4452328

[B32] Pöppel E. (1997). A hierarchical model of temporal perception. Trends Cogn. Sci. 1, 56–61. 10.1016/S1364-6613(97)01008-5 21223864

[B33] Scharnowski F. Rüter J. Jolij J. Hermens F. Kammer T. Herzog M. H. (2009). Long-lasting modulation of feature integration by transcranial magnetic stimulation. J. Vis. 9, 1–10. 10.1167/9.6.1 19761292

[B34] Schmidt H. McFarland J. Ahmed M. McDonald C. Elliott M. A. (2011). Low-level temporal coding impairments in psychosis: preliminary findings and recommendations for further studies. J. Abnorm. Psychol. 120, 476–482. 10.1037/a0023387 21553943

[B35] Shimojo S. (2014). Postdiction: its implications on visual awareness, hindsight, and sense of agency. Front. Psychol. 5:196. 10.3389/fpsyg.2014.00196 24744739 PMC3978293

[B36] Simon J. R. (1969). Reactions towards the source of stimulation. J. Exp. Psychol. 81, 174–176. 10.1037/h0027448 5812172

[B37] Singer W. (1993). Synchronization of cortical activity and its putative role in information processing and learning. Annu. Rev. Physiol. 55, 349–374. 10.1146/annurev.ph.55.030193.002025 8466179

[B38] Singer W. (1999). Neuronal synchrony: a versatile code for the definition of relations? Neuron 24, 49–65. 10.1016/S0896-6273(00)80821-1 10677026

[B39] Stroud J. M. (1955). The fine structure of psychological time, in Information Theory in Psychology: Problems and Methods ed. Quastler H. (Glencoe, IL: Free Press), 174–207.

[B40] Sweet A. L. (1953). Temporal discrimination by the human eye. Am. J. Psychol. 66, 185–198. 10.2307/1418725 13040525

[B41] Ullman S. (1979). The Interpretation of Visual Motion. Cambridge, MA: MIT Press.

[B42] van der Lubbe R. H. J. Abrahamse E. L. (2011). The premotor theory of attention and the Simon effect. Acta Psychol. 136, 259–264. 10.1016/j.actpsy.2010.09.007 20940067

[B43] van Rullen E. Zoefel B. Ilhan B. (2014). On the cyclic nature of perception in vision versus audition. Philos. Trans. R. Soc. Lond. B Biol. Sci. 369, 20130214. 10.1098/rstb.2013.0214 24639585 PMC3965168

[B44] van Wassenhove V. (2009). Minding time in an amodal representational space. Philos. Trans. R. Soc. Lond. B Biol. Sci. 364, 1815–1830. 10.1098/rstb.2009.0023 19487185 PMC2685822

[B45] Vatakis A. Spence C. (2007). Crossmodal binding: evaluating the “unity assumption” using audiovisual speech stimuli. Percept. Psychophys. 69, 744–756. 10.3758/BF03193776 17929697

[B46] Von Baer K. E. (ed.). (1864). Welche Auffassung der lebenden Natur ist die richtige? Und wie ist diese Auffassung auf die Entomologie anzuwenden?, in Reden, gehalten in wissenschaftlichen Versammlungen und kleinere Aufs4tze vermischten Inhalt, (the first part given as an oral lecture in 1860) (St. Petersburg: H. Schmitzdorf), 237–284.

[B47] Von Baer K. E. (1909). Welche Auffassung der lebenden Natur ist die richtige? Und wie ist diese Auffassung auf die Entomologie anzuwenden? Aus baltischer Geistesarbeit: Reden und Aufsätze 1, 1–47.

[B48] von Uexküll J. (1957). A stroll through the worlds of animals and men: a picture book of invisible worlds, Instinctive Behavior: The Development of a Modern Concept, ed. and trans. Schiller C. H. (New York: International Universities Press, Inc.), 5–80.

[B49] Wehrhahn C. Rapf D. (1992). ON- and OFF-pathways form separate neural substrates for motion perception: psychophysical evidence. J. Neurosci. 12, 2247–2250. 1607939 10.1523/JNEUROSCI.12-06-02247.1992 PMC6575930

[B50] Westheimer G. McKee S. P. (1977). Perception of temporal order in adjacent visual stimuli. Vis. Res. 17, 887–892. 10.1016/0042-6989(77)90062-1 595393

[B51] Whitney D. Murakami I. (1998). Latencydifference, not spatial extrapolation. Nat. Neurosci. 1, 656–657. 10.1038/3659 10196580

[B52] Wittmann M. (2011). Moments in time. Front. Integr. Neurosci. 5:66. 10.3389/fnint.2011.00066 22022310 PMC3196211

